# Limited social support is associated with depression, anxiety, and insomnia in a Japanese working population

**DOI:** 10.3389/fpubh.2022.981592

**Published:** 2022-11-21

**Authors:** Chie Omichi, Yuki Kaminishi, Hiroshi Kadotani, Yukiyoshi Sumi, Ayaka Ubara, Kohei Nishikawa, Arichika Matsuda, Yuji Ozeki

**Affiliations:** ^1^Department of Psychiatry, Shiga University of Medical Science, Otsu, Japan; ^2^Department of Hygiene and Public Health, Osaka Medical and Pharmaceutical University, Takatsuki, Japan; ^3^International Institute for Integrative Sleep Medicine (WPI-IIIS), University of Tsukuba, Tsukuba, Japan; ^4^Japan CBT Center, Hikone, Japan

**Keywords:** social support, job stress, depression, anxiety, insomnia, occupational health

## Abstract

**Background:**

Lack of social support is associated with depression, anxiety, and insomnia. This study aimed to determine the source of support related to depression, anxiety, and insomnia among Japanese workers.

**Methods:**

As part of a cohort study, we conducted a questionnaire survey among city government employees in Koka City, Shiga Prefecture, Japan, from September 2021 to March 2022. We used the Patient Health Questionnaire-9 (PHQ-9), Generalized Anxiety Disorder−7 (GAD-7), and Insomnia Severity Index (ISI) to assess depressive symptoms, anxiety symptoms, and insomnia, respectively. We used the Brief Job Stress Questionnaire (BJSQ) to assess job stressors and social support (from supervisors, colleagues, and family).

**Results:**

A total of 1,852 Japanese employees (38.4% male, 45.9 ± 12.9 years) participated in the survey, with 15.5, 10.8, and 8.2% of the participants having depressive symptoms (PHQ-9 ≥ 10), anxiety symptoms (GAD-7 ≥ 10), and insomnia (ISI ≥ 15), respectively. The logistic regression analysis suggested that job stressors were associated with depressive symptoms (*p* < 0.001), anxiety symptoms (*p* < 0.001), and insomnia (*p* = 0.009). In contrast, support from co-workers (*p* = 0.016) and family members (*p* = 0.001) was associated with decreased depressive symptoms. Support from family members was associated with decreased insomnia (*p* = 0.005).

**Conclusion:**

Social support from co-workers and family may be associated with reduced depressive symptoms, and family support may be associated with reduced insomnia in the Japanese working population.

**Clinical trial registration:**

https://clinicaltrials.gov/ct2/show/NCT03276585.

## Introduction

Stress in individual workers and the work environment has been reported to be associated with chronic absenteeism, turnover, suicide, and family disruption (1–3). A previous study reported that 40% of U.S. workers rated their jobs as very or extremely stressful and that 26% were often or very often burned out or stressed by their work (4). The National Institute for Occupational Safety and Health (NIOSH) defines occupational stress as “the harmful physical and emotional responses that occur when the requirements of the job do not match the capabilities, resources, or needs of the worker (4).” In the NIOSH model of job stress, stressful job conditions may lead to the risk of injury and illness, and individual and situational factors can modify and protect workers from risk (4).

Job stressors (exposure to stressful working conditions) may cause negative outcomes such as depression, anxiety, and insomnia (5). Depression is a major global public health problem and is projected to greatly contribute to disease burden worldwide in the coming decades (6, 7). Insomnia commonly occurs as a principal component of depression (8) and causes presenteeism (low work productivity due to being present at work, but ill or experiencing medical conditions) (9). Depression and anxiety are the two most prevalent mental disorders in the Japanese population (10, 11).

Social support is defined as a perception leading a person to believe that they are cared for and loved, esteemed, and a member of a network of mutual obligations (12). A systemic review indicated that low co-worker support and low supervisor support predicted the incidence of stress-related diseases, together with high job demands, low job control, low procedural justice, low relational justice, and a high effort-reward imbalance (13). A meta-analysis indicated that a high level of job stress, effort-reward imbalance, high demand, heavy workload, and low social support are associated with insomnia (14). Social support includes received and perceived social support. Received social support refers to the amount of support received, while perceived social support refers to its adequacy and availability (15). Research has shown that perceived social support is more closely related to mental health than received social support (16). Bidirectional associations were found between depressive and anxiety symptoms and loneliness (17). Loneliness was a stronger predictor of depressive and anxiety symptoms relative to the reverse causal direction. High loneliness may be a key risk factor for the development of future anxiety or depressive symptoms (17), thus, perceived social support not only from the workplace but also from family/friends may be important to decrease depression and anxiety levels in workplaces. According to a systemic review on depression and work-related risk factors, previous studies mainly reported support from supervisors and co-workers, but studies reporting support from family/friends were very limited (18). In another review of job stress, anxiety, and depression, family support was not mentioned (19). In a systemic review on insomnia and job stress, three studies reported social support, one reported support from supervisors or co-workers, and two did not report the origin of support (14).

Some studies analyzed the association between depression, anxiety, and insomnia among Japanese workers (20–24). Honda et al. reported that having little conversation with co-workers and/or supervisors was a risk factor for psychological distress among Japanese workers (20). Nishitani and Sakakibara reported that insomnia was related to the psychological response of depression in Japanese male workers of a manufacturing plant (21). Toyoshima et al. reported that insomnia directly affected state anxiety among Tokyo Medical University employees (22). Deguchi et al. reported that anxious temperament and role conflict were associated with insomnia among Japanese local government employees (23). Saojyo et al. found a synergistic association of job control and social support at work with depression and insomnia among Japanese government employees (24). However, these are cross-sectional studies, and depression, anxiety, insomnia, and social support (both at work and at home) were not simultaneously analyzed.

We hypothesized that lack of social support is associated with depressive symptoms, anxiety symptoms, and insomnia in the Japanese working population. The purpose of this study was to test this hypothesis and determine the source of support related to depression, anxiety, and insomnia among cohort study (NinJaSleep Study) participants.

## Materials and methods

### Participants

**Figure 1:**
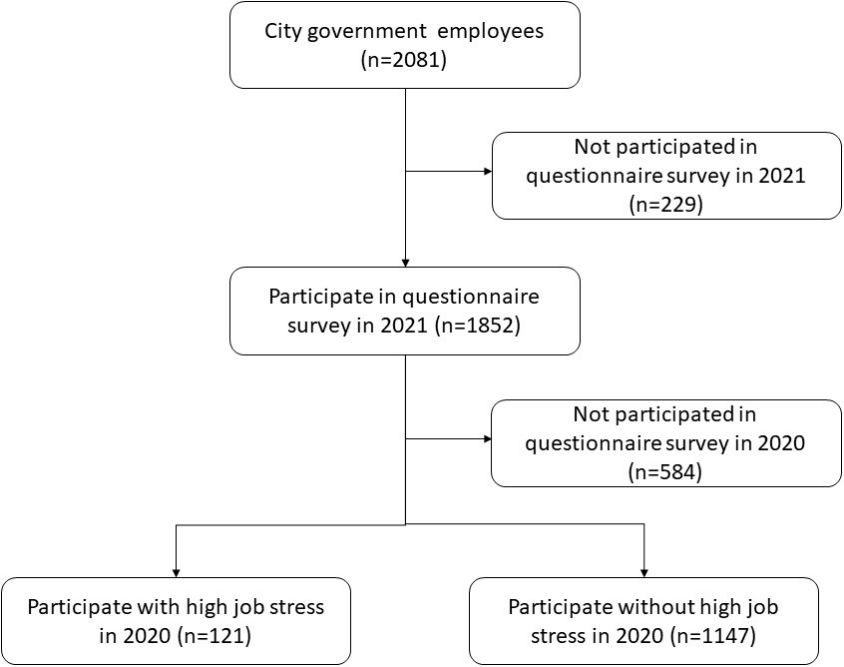
Flow diagram of the participant selection process.

We have performed a cohort study on sleep and mental health in a Japanese working population, named the Night in Japan Home Sleep Monitoring Study (NinJaSleep Study) (8, 25, 26). We conducted questionnaire surveys on sleep, mental health, and job stress among local government employees of Koka City, a rural city in Shiga Prefecture, Japan, which is known as the home of the ninja. Employees (*n* = 2081) were recruited for the questionnaire survey, and 1,852 employees participated in the survey from September 2021 to March 2022 (Figure 1).

The Ethics Committee of the Shiga University of Medical Science approved the study protocol (R2017–111). The study was registered at UMIN-CTR (UMIN000028675, registered on 2017/8/15) and ClinicalTrials.gov (NCT03276585, registered on 2017/9/3). Informed consent was obtained from all the participants. The datasets analyzed in this study are available from the corresponding author upon reasonable request.

### Questionnaire

Job stress was assessed using the Brief Job Stress Questionnaire (BJSQ) (16), a 57-item multidimensional job stress questionnaire evaluated on a Likert scale of 1 to 4. The BJSQ contains 17, 29, 9, and 2 items that assess job stressors, stress reactions, social support, and work/life satisfaction, respectively. Job stressors have nine subscales (job demands, job control, meaningfulness of work, work environment, suitability for work, physical burden, skill utilization, required job quality, and interpersonal relationships), with scores ranging from 17 to 68 and higher scores suggesting higher job stress levels.

“Social Support” included three subscales (supervisors, co-workers, and family), with scores ranging from 3 to 12 for each subscale and lower scores suggesting better support. “How freely can you talk with the following people?,” “How reliable are the following people when you are troubled?,” and “How well will the following people listen to you when you ask for advice on personal matters?” were asked about superiors, co-workers, and family (spouse, family, friends, etc.) on a four-point scale (1 = extremely, 2 = very much, 3 = somewhat, and 4 = not at all). The sum of the scores for superiors, co-workers, and family were separately calculated to indicate social support, with a range of 3–12 for each category. Cronbach's alpha coefficients for job demand, job control, and social support from supervisors, coworkers, and family/friends were reported to be 0.77–0.83, 0.68–0.69, 0.79–0.89, 0.76–0.85, and 0.83–0.86, respectively (27, 28). All BJSQ scales have been proven to have acceptable or high levels of internal consistency reliability and factor-based validity (27, 29). Workers with high job stress levels were identified using the BJSQ scoring program ver. 3.5 (Ministry of Health, Labor, and Welfare, Tokyo, Japan).

The Patient Health Questionnaire-9 (PHQ-9) is a 9-item questionnaire designed to screen for depression/depressive symptoms in clinical and research settings (30). The PHQ-9 contains items derived from the DSM-IV classification system pertaining to (1) anhedonia, (2) depressed mood, (3) trouble sleeping, (4) feeling tired, (5) change in appetite, (6) guilt or worthlessness, (7) trouble concentrating, (8) feeling slowed down or restless, and (9) suicidal thoughts; each item is scored from “0” (not at all) to “3” (nearly every day) (30). The standard cutoff score for screening to identify possible major depression/depressive symptoms is ≥ 10. In previous studies, participants with a PHQ-9 score ≥ 10 were classified as having depression (30, 31). According to a meta-analysis comparing PHQ-9 with validated diagnostic interviews, PHQ-9 had a sensitivity and specificity of 0.85 (95% confidence interval (CI):0.79–0.89) and 0.85 (95% CI: 0.82–0.87) to detect major depression, respectively (32).

The 7-item Generalized Anxiety Disorder Scale (GAD-7) is a widely used tool for assessing the frequency of anxiety symptoms in the past 2 weeks on a scale of “0” (not at all) to “3” (nearly every day) (33). The sum of the scores ranges from 0 to 21. Participants with GAD-7 scores ≥ 10 were classified as having moderate anxiety symptoms, indicating a need for further diagnostic testing (34).

Insomnia severity was measured using the Japanese version of the Insomnia Severity Index (ISI) (35), a validated 7-item self-report questionnaire that assesses insomnia severity over the past 3 weeks. The total score ranges from 0 to 28, with lower scores indicating fewer insomnia symptoms. Severity levels were categorized as no insomnia (0–7 points), sub-threshold (mild) insomnia (8–14 points), moderate insomnia (15–21 points), or severe insomnia (≥ 22 points) (36).

All these questionnaires were self-administered. Participants with symptoms of insomnia, anxiety, and depression were defined as having ISI ≥ 15, GAD-7 ≥ 10, and PHQ-9 ≥ 10, respectively.

Demographic data, including birth year and month, height (cm), body weight (kg), sex, and history of chronic conditions such as hypertension, diabetes, and lipidemia were also collected. Age in September 2021 was calculated from birth year and month; BMI was calculated from height and body weight; and history of diagnosis (yes/no) of hypertension, diabetes, and lipidemia were asked.

### Statistical analysis

We compared proportions between groups using the χ^2^ test and analyzed continuous data using the *t*-test.

We performed a logistic regression analysis with depressive symptoms (PHQ-9 ≥ 10), anxiety symptoms (GAD-7 ≥ 10), and insomnia (ISI ≥ 15) as dependent variables after adjusting for age, sex, BMI, job stressors, support from supervisors, support from co-workers, support from family, and high job stress. The results of high job stress analysis were assessed using the BJSQ in the previous year for two reasons. First, since the BJSQ determines high stress based on job stressor and social support, it may be not appropriate to analyze correlations using high stress determinations from the same year. Second, after high job stress was detected, workers who experienced this were expected to be cared for by their supervisors, co-workers, and family. Thus, the high job stress in the previous year may have affected their support.

We performed a linear regression analysis as a sensitivity analysis using the PHQ-9, GAD-7, or ISI scores as dependent values. All models included job stressors, and support from supervisors, co-workers, and family.

All data were analyzed using SPSS 25.0, statistical software (SPSS Inc., Chicago, IL), and MedCalc ver. 20.106 (MedCalc Software Ltd., Ostend, Belgium). Differences were considered statistically significant at *p* < 0.05.

## Results

The participation rate was 89.0% (1852/2081). Among the eligible participants, 38.4 and 61.6% were males and females, respectively (Table 1). Furthermore, 15.5, 10.8, and 8.2 of the participants had depression (PHQ-9 ≥ 10), anxiety (GAD-7 ≥ 10), and insomnia (ISI ≥ 15), respectively (Table 1). Of the participants, 41.8, 52.4, 5.7, and 0.1% classified their occupation as administrative/clerical, educational/teaching, medical/health support, or temporary/contracted, respectively. Of these jobs, 2.8% were night shifts.

**Table 1:** Characteristics of participants.

**Number**		**1852**
Age	Years	45.9 ± 12.9
Sex	Male n(%)	712 (38.4)
BMI	kg/m^**2**^	22.6 ± 3.65
PHQ-9		4.85 ± 4.78
	PHQ-9 ≥ 10, %	15.5
GAD-7		3.83 ± 4.22
	GAD-7 ≥ 10, %	10.8
ISI		7.32 ± 4.77
	ISI ≥ 15, %	8.2
Hypertension	***n*** (%)	251 (13.6)
Diabetes	***n*** (%)	64 (3.46)
Lipidemia	***n*** (%)	164 (8.85)
		
With high job stress	%	9.5

High job stress assessed with BJSQ in 2021. Mean ± standard deviation.

PHQ-9, Patient Health questionnaire-9; GAD-7, 7-item Generalized Anxiety Disorder Scale; ISI, insomnia severity index.

Job stressors and support from supervisors, co-workers, and family members were significantly associated with depressive symptoms, anxiety symptoms, and insomnia (Table 2). We further analyzed the association between depressive symptoms, anxiety symptoms, insomnia, job stressors, and social support using logistic regression analysis (Table 3). Job stressors as well as lack of support from supervisors, co-workers, and family were associated with depressive symptoms, anxiety symptoms, and insomnia in the unadjusted model. In the adjusted model, job stressors were significantly associated with depressive and anxiety symptoms and insomnia. Support from co-workers and family members was associated with depressive symptoms, and family support was associated with insomnia. For sensitivity analysis, we performed a linear regression analysis. The results of the linear regression analysis were like those of the adjusted logistic regression analysis (Table 4).

**Table 2:** Comparison between participants with and without depressive symptoms, anxiety symptoms, and insomnia.

	**PHQ-9 < 10**	**PHQ-9 ≥10**	***p-*value**	**GAD-7 < 10**	**GAD-7 ≥10**	***p-*value**	**ISI < 15**	**ISI ≥15**	***p-*value**
*N* (%)	1565 (84.5)	287 (15.5)		1652 (89.2)	200 (10.8)		1701 (91.8)	151 (8.15)	
Age, years	46.6 ± 13.1	42.2 ± 11.3	**<** **0.001**	46.4 ± 13.1	41.7 ± 10.9	**<** **0.001**	46.1 ± 13.1	44.3 ± 11.2	0.062
Sex, male%	38.8	36.6	0.509	39.0	34.0	0.191	38.2	41.7	0.384
BMI, kg/m^2^	22.6 ± 3.59	22.6 ± 3.95	0.952	22.6 ± 3.63	22.5 ± 3.81	0.717	22.6 ± 3.61	22.7 ± 4.09	0.612
Job stressors	41.4 ± 6.83	46.7 ± 8.98	**<** **0.001**	41.5 ± 6.89	47.6 ± 6.70	**<** **0.001**	41.84 ± 6.99	46.1 ± 7.46	**<** **0.001**
Support from supervisors	6.89 ± 2.08	7.99 ± 2.26	**<** **0.001**	6.95 ± 2.08	7.94 ± 2.40	**<** **0.001**	6.99 ± 2.11	7.83 ± 2.37	**<** **0.001**
Support from co-workers	6.54 ± 1.99	7.65 ± 2.12	**<** **0.001**	6.61 ± 2.01	7.52 ± 2.22	**<** **0.001**	6.63 ± 2.01	7.57 ± 2.24	**<** **0.001**
Support from family	5.04 ± 1.94	6.00 ± 2.32	**<** **0.001**	5.14 ± 1.99	5.61 ± 2.31	**<** **0.001**	5.12 ± 1.99	6.03 ± 2.32	**<** **0.001**
Hypertension	227 (14.6)	24 (8.4)	**0.005**	234 (14.3)	17 (8.5)	**0.026**	231 (13.7)	20 (13.2)	0.876
Diabetes	62 (4.0)	2 (0.7)	**0.005**	62 (3.8)	2 (1.0)	**0.043**	62 (3.7)	2 (1.3)	0.131
Lipidemia	141 (9.1)	23 (8.1)	0.579	146 (8.9)	18 (9.0)	0.953	149 (8.8)	15 (9.9)	0.652

Job stressors and support were assessed using the brief job stress questionnaire. Mean ± standard deviation (SD).

PHQ-9, Patient Health questionnaire-9; GAD-7, 7-item Generalized Anxiety Disorder Scale; ISI, insomnia severity index. P-values < 0.05 were indicated in bold. Percentages for hypertension, diabetes and lipidemia represent percentages in the respective columns.

**Table 3:** Logistic regression analysis of depressive symptoms, anxiety symptoms, and insomnia.

	**Unadjusted**			**Adjusted**		
	**OR**	**(95% CI)**	***p-*value**	**OR**	**(95% CI)**	***p-*value**
**Depressive symptoms (PHQ-9 ≥10)**						
Job stressors	1.124	(1.101–1.147)	**<** **0.001**	0.080	(1.048–1.114)	**<** **0.001**
Support from supervisors	1.289	(1.210–1.374)	**<** **0.001**	0.997	(0.886–1.121)	0.957
Support from co-workers	1.324	(1.239–1.416)	**<** **0.001**	1.163	(1.029–1.315)	**0.016**
Support from family	1.246	(1.173–1.323)	**<** **0.001**	1.164	(1.067–1.271)	**0.001**
						
**Anxiety Symptoms (GAD-7** **≥10)**	**Unadjusted**			**Adjusted**		
	OR	95% CI	*p-*value	OR	95% CI	*p-*value
Job stressors	1.141	(1.114–1.168)	**<** **0.001**	1.098	(1.061- 1.137)	**<** **0.001**
Support from supervisors	1.252	(1.164–1.347)	**<** **0.001**	1.028	(0.904–1.170)	0.674
Support from co-workers	1.251	(1.160–1.349)	**<** **0.001**	1.082	(0.946- 1.238)	0.249
Support from family	1.116	(1.040–1.196)	**0.002**	0.999	(0.903–1.104)	0.982
						
**Insomnia (ISI** **≥15)**	**Unadjusted**			**Adjusted**		
	OR	95% CI	*p-*value	OR	95% CI	*p-*value
Job stressors	1.092	(1.065–1.119)	**<** **0.001**	1.050	(1.012–1.088)	**0.009**
Support from supervisors	1.207	(1.113–1.310)	**<** **0.001**	1.031	(0.894–1.189)	0.677
Support from co-workers	1.260	(1.157–1.373)	**<** **0.001**	1.077	(0.927–1.251)	0.330
Support from family	1.225	(1.134–1.324)	**<** **0.001**	1.164	(1.047–1.293)	**0.005**

Adjusted for age, sex, BMI, job stressors, support from supervisors, support from co-workers, support from family, hypertension, diabetes, lipidemia, and high job stress in the previous year. Job stressors and support were assessed using the Brief Job Stress Questionnaire during the same year.

PHQ-9, Patient Health questionnaire-9; GAD-7, 7-item Generalized Anxiety Disorder Scale; ISI, insomnia severity index. P-values < 0.05 were indicated in bold.

**Table 4:** Linear regression analysis of PHQ-9, GAD-7, and ISI.

**PHQ-9**	**Total**	
	β	***p-***value
Job stressors	0.346	**< 0.001**
Support from supervisors	0.052	0.089
Support from co-workers	0.066	**0.033**
Support from family	0.149	**< 0.001**
		
GAD-7	Total	
	β	***p-***value
Job stressors	0.379	**< 0.001**
Support from supervisors	0.025	0.405
Support from co-workers	0.072	**0.020**
Support from family	0.088	**< 0.001**
		
ISI	Total	
	β	***p-***value
Job stressors	0.253	**< 0.001**
Support from supervisors	0.061	0.056
Support from co-workers	0.026	0.419
Support from family	0.145	**< 0.001**

Job stressors and support from supervisors, co-workers, and family members were assessed using the brief job stress questionnaire.

PHQ-9, patient health questionnaire-9; GAD-7, 7-item Generalized Anxiety Disorder scale; ISI, insomnia severity index. P-values < 0.05 were indicated in bold.

**Figure 2:**
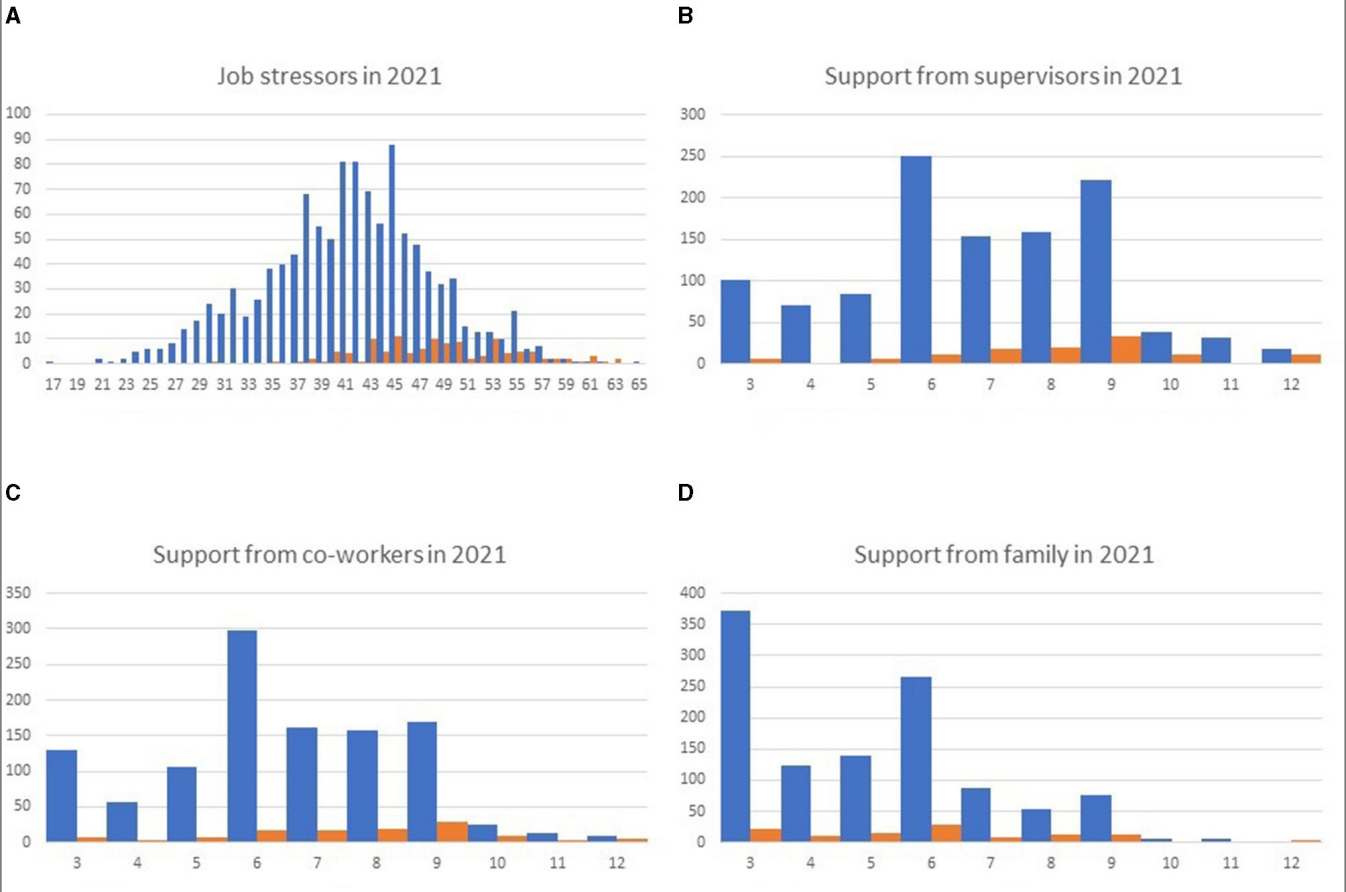
Histogram of stressors and support scores in 2021, classified according to the presence (orange) or absence (blue) of high stress levels in 2020. Job stressors **(A)** and support from supervisors **(B)**, co–workers **(C)**, and family **(D)** are presented. A smaller number of job stressors suggests less job stress. Smaller scores in the responses to the questions about support suggest a better support situation.

When workers had high job stress levels, job stressors decreased and support from supervisors and co-workers improved in the following year (Table 5). However, support from the family did not change. When the distribution of participants with and without high job stress levels in 2020 was analyzed separately, apparent differences were observed in job stressors, support from supervisors, and support from co-workers (Figure 2).

**Table 5:** Changes in job stressors and social supports according to high job stress.

	**With high job stress**	**Without high job stress**	***p–*value**
Delta job stressors	−1.61 ± 5.98	1.13 ± 5.54	**< 0.001**
Delta support from supervisors	−0.47 ± 2.23	0.08 ± 2.03	**0.011**
Delta support from co–workers	−0.52 ± 2.19	0.10 ± 1.88	**0.003**
Delta support from family	0.01 ± 1.72	0.18 ± 1.70	0.297

Mean ± standard deviation (SD). The changes in scores from 2020 to 2021 were compared with those of high job stress in 2020. A smaller number of job stressors suggests less job stress. A smaller number of supports suggests better support. Job stressors and support from supervisors, co–workers, and family members were assessed using the brief job stress questionnaire. P-values < 0.05 were indicated in bold.

## Discussion

A questionnaire survey was conducted to analyze the association between social support and mental health outcomes in the Japanese working population. Higher scores on social support in the BJSQ (suggesting poorer support) were associated with higher PHQ-9, GAD-7, and ISI scores (suggesting severe symptoms of depression, anxiety, and insomnia, respectively) (Tables 2, 3). High job demand (increased workload/time pressure), low job control (minimal decision-making), and low social support have been reported to be associated with poorer employee mental health (37) and insomnia (38). Thus, social support may be important for preventing mental health problems in the working population.

The development of the BJSQ was based on the NIOSH job stress model (39). In the BJSQ, job stressors may include stressful job conditions, and social support may suggest individual and situational factors in the model (40). Job stressors were significantly associated with depression, anxiety, and insomnia in all analyses. Higher social support scores, which suggest poorer support, were associated with worse outcomes (higher PHQ-9, GAD-7, and ISI scores). These results may fit well with the NIOSH job stress model (4).

We found that lack of support from co-workers and family was associated with depressive symptoms and that lack of support from family was associated with insomnia. Support from closer groups seems to work protectively against depression and insomnia.

Comparing the presence of high stress levels in the previous year with changes in workplace stressors and social support in that year and the following year, the high stress level group showed a decrease in workplace stressors and improved support from supervisors and coworkers. This suggests that the high stress level-related decisions led to an improvement in the work environment in the following year.

In the previous year's high stress level group, the degree of family support did not change between that year and the following year. On the other hand, a logistic regression analysis of the relationship between family support and outcomes such as depression and insomnia in the same year showed a significant correlation. It is possible that family support may have already been provided sufficiently when the patient was determined to be highly stressed in the previous year.

Stressors and social support were compared in histograms stratified by the presence or absence of high stress levels in the previous year. The distribution of family support showed no clear relationship with the presence or absence of high stress levels in the previous year. On the other hand, the distribution of stressors and support from supervisors and co-workers apparently differed depending on the presence or absence of high stress levels in the previous year. These results suggest that family members may have already provided sufficient support to those in the high stress level group in the previous year and that although support from supervisors and co-workers improved after the high stress level rating, there may be still room for further improvement.

A systematic review of human resource management training programs aimed at teaching supervisors how to reduce employees' psychosocial stress reported only poor quality and inconsistent results (39). Some studies have suggested that supervisor training may be beneficial, but others have shown no improvement when compared with the absence of intervention (39). Both in-person (face-to-face) (41) and computer-based (web- and mobile-based) stress-management interventions (42) have reportedly been effective in reducing job stress levels. In this study, support from supervisors improved between the first and second years of the study but was not significantly associated with employee outcomes. However, a reduction in job stress levels was associated with decreased depression, anxiety, and insomnia. Stress-management interventions and reduction in job stressors may be more effective strategies than supervisor education to reduce workplace stress. We found that family support protects against depression and insomnia.

This study had some limitations. Our target population was government employees in a rural city in Japan. Our results cannot be generalized to other parts of Japan or other countries without further investigations. We plan to use the results of this study in our activities as occupational physicians to provide interventions aimed at reducing long-term absence and turnover through improved social support in multiple workplaces. This was a questionnaire survey, and structured interviews were not performed to diagnose depression, anxiety, insomnia, or other mental disorders. Social support was also assessed using a questionnaire asking how the participants recognized support but did not ask how much support was provided. However, perceived social support has been reported to have significantly higher effects than actual social support on promoting mental health (43).

The results of this cohort study have been displayed as posters in the cafeteria of the City Hall and lectures have been held to publicize the results to the participants (25). We plan to publicize the results of the present study in the same manner. We believe that incorporating into workplace training programs the usefulness of social support from supervisors and coworkers in improving depression, anxiety disorders, and insomnia will be useful in preventing these problems in the workplace.

In conclusion, social support from co-workers and family may be associated with decreased depressive symptoms, and family support may be associated with decreased insomnia in the Japanese working population. Anxiety symptoms were mainly associated with job stressors.

## Data availability statement

The datasets analyzed in this study are available from the corresponding author upon reasonable request.

## Ethics statement

The studies involving human participants were reviewed and approved by the Ethics Committee of the Shiga University of Medical Science. The patients/participants provided their written informed consent to participate in this study.

## Author contributions

Conceptualization: YK and HK. Methodology, software, formal analysis, writing original draft preparation, visualization, supervision, and funding acquisition: HK. Validation: YS. Investigation: CO and HK. Resources: KN and HK. Data acquisition: the NinJaSleep Study Group. Data curation: CO. Writing review and editing: YK, CO, YS, AU, KN, AM, and YO. Project administration: YO. All authors have read and agreed to the published version of the manuscript.

## Funding

This work was supported in part by a research grant from the Investigator-Initiated Studies Program of Merck Sharp & Dohme LLC/MSD K.K. HK received grants from Eisai Co., Ltd., (HHCS20210930005) and SECOM Science and Technology Foundation. HK reported consulting fees from Takeda Pharmaceutical Co. Ltd. HK, AU, and AM were associated with a laboratory that was supported by donations from Fukuda Lifetech Co., Ltd., and Fukuda Life Tech Keiji Co., Ltd., to the Shiga University of Medical Science. JSPS KAKENHI grant number 22K18384.

## Conflict of interest

This work was supported in part by a research grant from the Investigator-Initiated Studies Program of Merck Sharp & Dohme LLC/MSD K.K. The opinions expressed in this paper are those of the authors and do not necessarily represent those of Merck Sharp & Dohme LLC / MSD K.K. HK received grants from Eisai Co., Ltd., and the SECOM Science and Technology Foundation. HK reports consulting fees from Takeda Pharmaceutical Co., Ltd., HK, AU, and AM were associated with a laboratory that was supported by donations from Fukuda Lifetech Co., Ltd., and Fukuda Life Tech Keiji Co., Ltd., to Shiga University of Medical Science. The funders had no role in the design of the study; in the collection, analyses, or interpretation of data; in the writing of the manuscript, or in the decision to publish the results. JSPS KAKENHI grant number 22K18384. The remaining authors declare that the research was conducted in the absence of any commercial or financial relationships that could be construed as a potential conflict of interest.

## Publisher's note

All claims expressed in this article are solely those of the authors and do not necessarily represent those of their affiliated organizations, or those of the publisher, the editors and the reviewers. Any product that may be evaluated in this article, or claim that may be made by its manufacturer, is not guaranteed or endorsed by the publisher.
