# Corrigendum: Limited social support is associated with depression, anxiety, and insomnia in a Japanese working population

**DOI:** 10.3389/fpubh.2024.1449081

**Published:** 2024-07-01

**Authors:** Chie Omichi, Yuki Kaminishi, Hiroshi Kadotani, Yukiyoshi Sumi, Ayaka Ubara, Kohei Nishikawa, Arichika Matsuda, Yuji Ozeki

**Affiliations:** ^1^Department of Psychiatry, Shiga University of Medical Science, Otsu, Japan; ^2^Department of Hygiene and Public Health, Osaka Medical and Pharmaceutical University, Takatsuki, Japan; ^3^International Institute for Integrative Sleep Medicine (WPI-IIIS), University of Tsukuba, Tsukuba, Japan; ^4^Japan CBT Center, Hikone, Japan

**Keywords:** social support, job stress, depression, anxiety, insomnia, occupational health

In the published article, there was an error in the legends within Figure 2. The corrected Figure 2 and its caption appear below.

**Figure 2:**
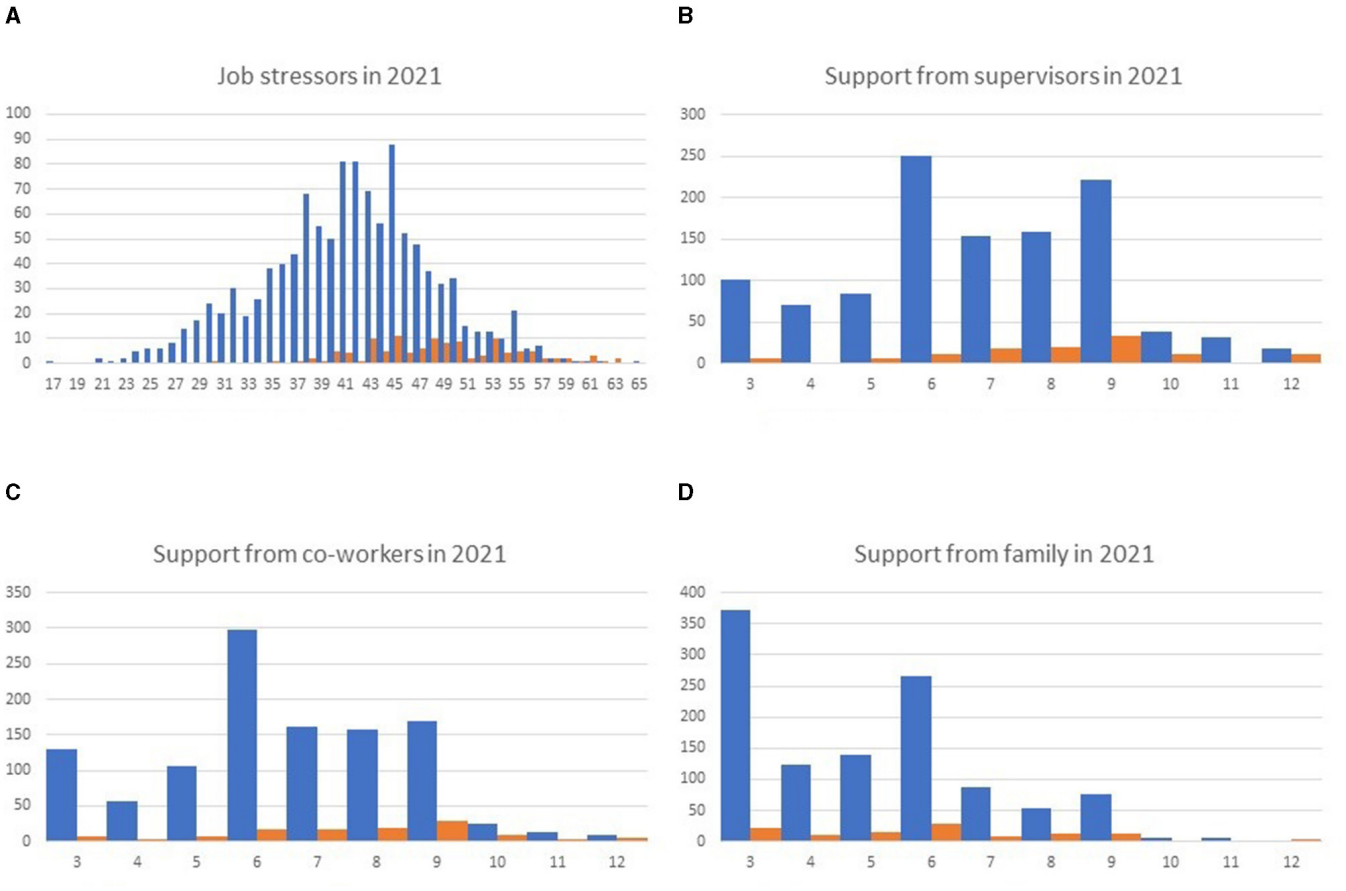
Histogram of stressors and support scores in 2021, classified according to the presence (orange) or absence (blue) of high stress levels in 2020. Job stressors **(A)** and support from supervisors **(B)**, co–workers **(C)**, and family **(D)** are presented. A smaller number of job stressors suggests less job stress. Smaller scores in the responses to the questions about support suggest a better support situation.

The authors apologize for this error and state that this does not change the scientific conclusions of the article in any way. The original article has been updated.

